# A Simple Spectrophotometric Method for Determination of Glyoxylic Acid in Its Synthesis Mixture

**DOI:** 10.1155/2020/5417549

**Published:** 2020-03-19

**Authors:** Mazhar Abdulwahed, Lamia Mamoly, Wael Bosnali

**Affiliations:** Damascus University, Faculty of Sciences, Damascus, Syria

## Abstract

A new simple and reliable spectrophotometric method is described to determine glyoxylic acid in its synthesis reaction mixture containing oxalic acid, glycolic acid, acetic acid, glyoxal, and ethylene glycol by means of a modified Hopkins–Cole reaction between glyoxylic acid and tryptophan in presence of ferric chloride and concentrated sulphuric acid. The linear range of glyoxylic acid concentration is 0–0.028 M. The limits of detection (LOD) and quantitation (LOQ) are 0.0019 M and 0.00577 M, respectively. The LOD, LOQ, standard deviation, relative standard deviation, and recovery ratio of the proposed method are comparable with a selected HPLC reference method. Both methods displayed same precision and credibility. Reaction stoichiometry between tryptophan and glyoxylic acid is assumed to be 2 : 3. Reaction mechanism has been postulated based on identified molar ratios of reactants. Glyoxal gave a negative test with tryptophan although it is a dialdehyde.

## 1. Introduction

Glyoxylic acid (GA) is an important organic acid in the chemical, cosmetic, pharmaceutical, and food industries. It is found in plants and involved in the metabolic cycle of animals. Glyoxylic acid is produced in several ways: by nitric acid oxidation of glyoxal, by catalytic oxidation of ethylene or acetaldehyde, and by electrochemical reduction of oxalic acid [1].

The determination of glyoxylic acid in its electrochemical synthesis reaction mixture is a difficult process because this mixture contains carboxylic acids with convergent acidic dissociation constants and other compounds carrying organic groups that can undergo oxidation or reduction such as ethylene glycol, glyoxal, and glycolic acid. Thus, it is expected that glyoxylic acid will be very difficult to determine quantitatively using traditional analytical methods such as acid-base or redox titrations, separation, or precipitation [2, 3].

This paper provides a simple, reliable, and inexpensive spectrophotometric method for determination of glyoxylic acid in such reaction mixtures directly without the need of separation. The method depends on a colored chromogen product formation between glyoxylic acid and tryptophan when they react according to a reaction discovered in 1901 by Hopkins and Cole [4] (see equation (1)). They suggested that one molecule of glyoxylic acid can react with two molecules of tryptophan to give a crimson-violet color that absorbs at 540–545 nm. This reaction ideally occurs in an anhydrous medium pledged by concentrated sulphuric acid [5–8]:(1)
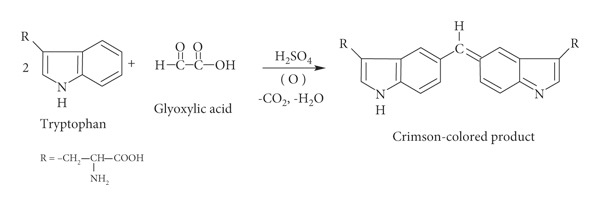



Since its discovery, the Hopkins–Cole test has been neither easy nor reliable for tryptophan quantitative measurements due to slow rate of product formation, various color evolutions, and low stability of the product. This pushed copious researchers to suggest many improvements as in [9–17]. In [9, 10], auxiliary materials such as copper sulphate were added that gave a dark crimson-violet color product. Cary [9] was the pioneer in the description of the crimson-violet color absorption at 560 nm. Other researchers [11–14] studied the compounds and stoichiometry that can form between organic aldehydes and tryptophan. Quantitative determination of tryptophan in proteins succeeded by addition of mild oxidants such as persulfate and Fe^3+^ ions, which accelerated chromogen appearance and increased its stability [15–17]. However, the test was not satisfactory for quantitative glyoxylic acid determination so far, eventually due to the factors affecting its accuracy as to be elucidated in the context of this article.

Glyoxylic acid spectrophotometric analysis has been also used to determine enzyme activity by a color reaction with 2-aminobenzaldehyde and glycine [18] or by forming a color product with phenylhydrazine, but this method can be affected by the presence of other aldehydes in the mixture [19].

Determination of glyoxylic acid in similar organic mixtures using the HPLC technique has been successful in [20, 21] using refractive index and photo diode array detectors, respectively. Another method required the conversion of glyoxylic acid into one of its derivatives with 2,4-dinitrophenylhydrazine [22, 23]. Glyoxylic acid in other mixtures was also determined by this technique however using a more sensitive detector, the fluorescence detector, after converting glyoxylic acid into a fluorocarbon derivative with a detection limit reaching 5 nanomol/L [24].

Other methods such as differential pulse polarography technique [25] and ion chromatography technology [26] have been likewise used to analyze similar reaction mixture with diverse degrees of success.

The aim of this work is to provide a new rapid, reliable, and inexpensive spectrophotometric method for determination of glyoxylic acid in electrochemical synthesis mixture directly without the need of separation.

### 1.1. Materials

Oxalic acid (BDH) 99%, glyoxylic acid (Merck) water solution 50%, glycolic acid (Sigma Aldrich) 99%, glyoxal (Merck) water solution 40%, ethylene glycol (BDH) 99.5%, glacial acetic acid (SDFCL) 99.5%, L-tryptophan (BDH) 93%, sulphuric acid (Panreac) 95–98%, ferric chloride hexahydrate (Avonchem) 98% fresh packaging were purchased.

### 1.2. Instruments

Spectrophotometer (721-2000), Caihong Corporation Limited, and HPLC column: Knauer Eurokat columns H Form, RI detector (Knauer), were used.

### 1.3. Experimental Work

The reaction of Hopkins and Cole (equation (1)) was applied on each component of the reaction mixture consisting of oxalic acid, glyoxylic acid, glycolic acid, glyoxal, ethylene glycol, and acetic acid solution in water at 0.028 M concentration in the following procedure.

To each individual test tube, place 0.25 mL of the reactant solution, 0.6 mL of tryptophan 0.016 M solution, and 2 mL of fresh ferric chloride 0.025 M solution. Mix thoroughly and then pour 5 mL of concentrated sulphuric acid divided in around 1 mL portions over 30 minutes under continuous stirring in a cold bath of running tap water to prevent any increase in temperature beyond 50°C. Then, read absorbance at 560 nm within 10–20 minutes. If to allow samples to stand the elongated time, secondary transformations involving the Fe^+2^ ion might cause a color change and test failure.

### 1.4. Results and Discussion

#### 1.4.1. Applicability of the Method for Quantitative Determination of Glyoxylic Acid in Mixture

**Figure 1:**
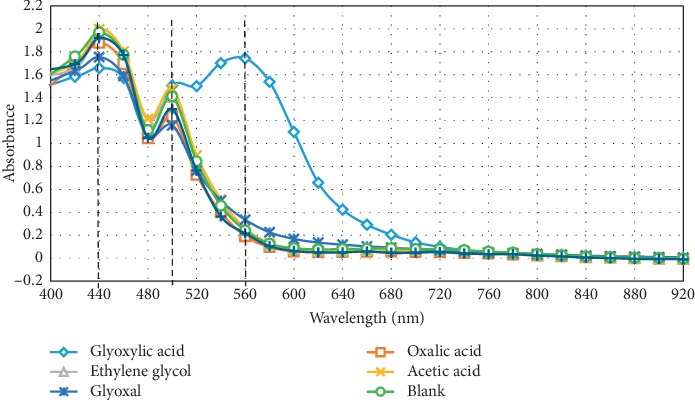
Optical absorption spectrum of reaction product between each component of the studied mixture and tryptophan.

Figure 1 shows the spectra obtained using the proposed test procedure on glyoxylic acid and other mixture constituents in addition to a blank sample. Water replaced the organic compound in the blank sample.

Three absorption maxima can be observed at wavelengths 440, 500, and 560 nm. The maximum at 560 nm is a characteristic for glyoxylic acid. The other two maxima are common for all compounds, including the blank sample. Thus, they can be attributed to the interaction with ferric chloride (see Section 2.2). Accordingly, the Hopkins–Cole reaction test by means of the proposed method is selective only to glyoxylic acid without any interference of other compounds of the studied mixture including the glyoxal containing aldehyde group. The baseline, however, has to be zero set using the blank sample in order to compensate the minor effect of ferric chloride interaction with tryptophan.

**Figure 2:**
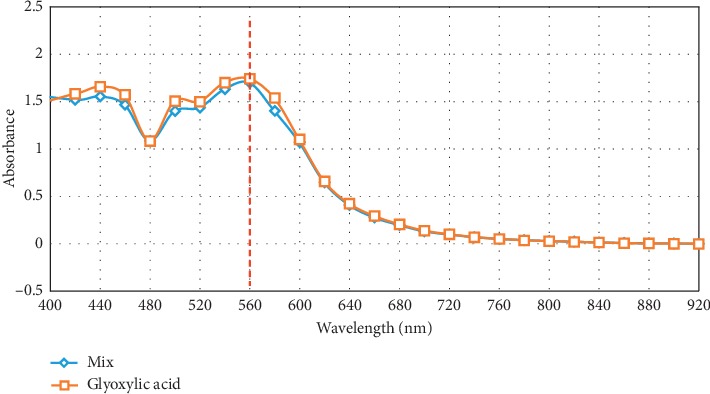
Optical absorption spectrum of a mixture containing oxalic acid, glyoxylic acid, glycolic acid, glyoxal, ethylene glycol, and acetic acid in comparison with pure glyoxylic acid, each at a concentration of 0.028 M after a reaction with tryptophan in presence of ferric chloride and concentrated sulphuric acid.

Furthermore, Figure 2 depicts the spectra obtained for 0.028 M glyoxylic acid solution alone and 0.028 M glyoxylic acid in mixture solution using the proposed test procedure. It can be clearly seen that both pure glyoxylic acid and the glyoxylic acid in the mixture exhibit practically the same absorbance at 560 nm.

## 2. Optimization of the Method Variables

The process of the optimization aims to make the Hopkins–Cole reaction only related to the concentration of glyoxylic acid in the reaction mixture.

### 2.1. Influence of Concentrated Sulphuric Acid

**Figure 3:**
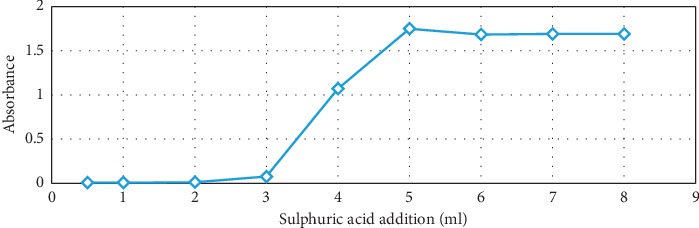
Optical absorption versus volume of concentrated sulfuric acid in the reagent with 0.25 mL glyoxylic acid 0.028 M, 0.6 mL tryptophan 0.016 M, and 2 mL of ferric chloride 0.025 M.

Added volume of concentrated sulphuric acid was varied: 0.5, 1, 2, 3, 4, 5, 6, 7, and 8 mL, while amounts of reactants were kept constant at 0.25 mL glyoxylic acid 0.028 M solution, 0.6 mL tryptophan 0.016 M solution, and 2 mL ferric chloride 0.025 M solution. The final volume of liquid in tubes was corrected with distilled water. Test procedure was described in the experimental work section.  Figure 3 shows an increase in optical absorbance up to 5 mL sulphuric acid addition, which means that sufficient amount of concentrated sulphuric acid must be present in order to absorb reaction and hydration water. Otherwise, the aldehyde group of glyoxylic acid may be hydrated to geminal diol (*K* = 300 at 25°C) and eventually further dimerized to hemiacetal, thus making it not be available for reaction with tryptophan.

### 2.2. Influence of Ferric Chloride

**Figure 4:**
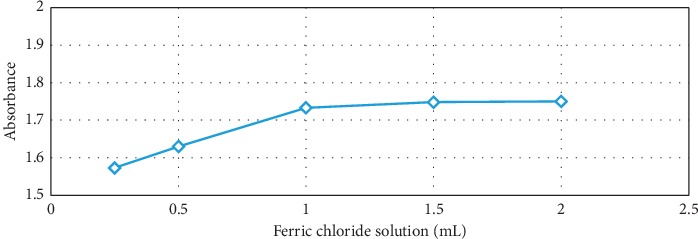
Optical absorption versus volume of ferric chloride in the reagent at 0.25 mL glyoxylic acid 0.028 M and 0.6 ml tryptophan 0.016 M.

The added volume of ferric chloride 0.025 M solution was varied: 0.25, 0.5, 1, 1.5, and 2 mL, while the other mixture constituents and test procedure remained unchanged (see Section 2.1).  Figure 4 shows a proportional increase in absorbance with increasing ferric chloride solution volume up to 1 mL, followed by a slight further increase up to 2 mL ferric chloride addition. Thus, the oxidizing agent needs to be present in the reaction mixture in sufficient amount for satisfactory performance.

**Figure 5:**
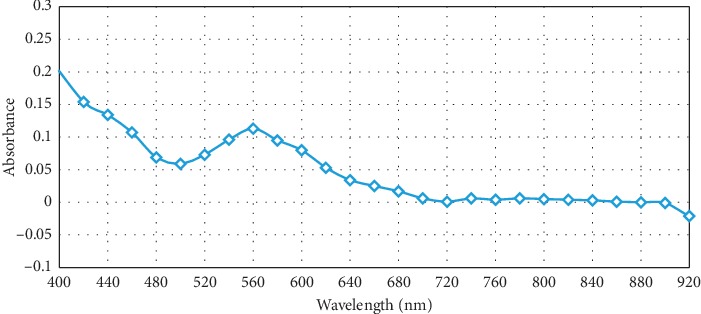
Optical absorption spectrum for reaction product of glyoxylic acid and tryptophan 0.016 M in presence of concentrated sulfuric acid and absence of ferric chloride.

To confirm its responsibility for absorption maxima at 440 and 500 nm in Figures 1 and 2, a separate test has been run without addition of ferric chloride solution.  Figure 5 displays the disappearance of these maxima. However, the maximum at 560 nm, which is representative for glyoxylic acid, showed far less intensity than that shown in Figure 1. It hence demonstrates that ferric chloride, in agreement with [16, 17], increases the yield of pigment product.

**Figure 6:**
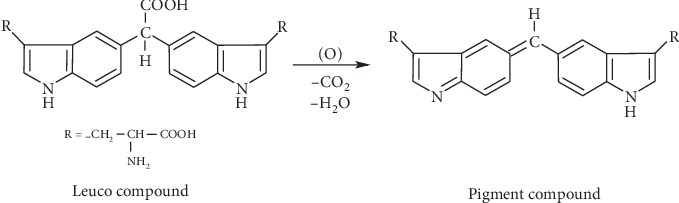
Proposed reaction from leuco-to-pigment compound under the effect of iron ion as an oxidizing agent, concurring Fearon [13].

The mechanism of the iron III ion effect can be ascribed to oxidation reaction in the leuco dye molecule, which results from condensation of two tryptophan molecules with one glyoxylic acid molecule (see Figure 6 and Section 4).

### 2.3. Influence of Tryptophan

**Figure 7:**
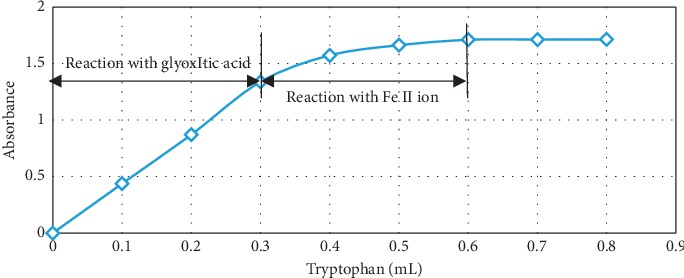
Optical absorption versus volume of tryptophan 0.016 M solution in the reagent at 0.25 mL glyoxylic acid 0.028 M, 2 mL ferric chloride 0.025 M, and 5 mL concentrated sulfuric acid.

The volume of tryptophan 0.016 M solution was varied: 0.1, 0.2, 0.25, 0.3, 0.4, 0.6, 0.8, and 1.0 mL, while other mixture constituents and test procedure remained unchanged (see Section 2.1). As can be seen in Figure 7, there is a proportional increase in absorbance with increased added volume of tryptophan solution until reaching 0.3 mL. At this point, tryptophan amount (0.48 × 10^−5^ moles) should have reacted with the complete amount of glyoxylic acid present in the sample (amounting to 0.7 × 10^−5^ moles). Afterwards, absorbance increased sluggishly nearing a plateau at 0.6 mL (or 0.6 *∗* 0.016 = 0.96 *∗* 10^−5^ mol). The later increase in absorbance with a larger tryptophan solution volume can be ascribed to the interaction between tryptophan and iron II ion produced during reaction. We decided to keep the amount of tryptophan in excess in all upcoming tests to compensate for its loss in reaction with the iron II ion.

### 2.4. Influence of Temperature

Numerous tests were conducted (not shown here) at different temperatures ranging from ambient to 90°C. At ambient temperature, it was practically difficult to conduct a successful test due to rapid and intense heat generation by the reaction itself and by sulphuric acid hydration. However, tests carried out above 50°C produced differing colors from crimson-violet and lacked reproducibility. This is not astonishing because tryptophan-glyoxylic acid reds are known to be greatest unstable pigments. They readily lose CO_2_ and soon oxidize on little warming [13]. Fortunately, tests conducted at temperatures 48–50°C exhibited very good reproducibility and formed stable color.

## 3. The Standard Curve

**Figure 8:**
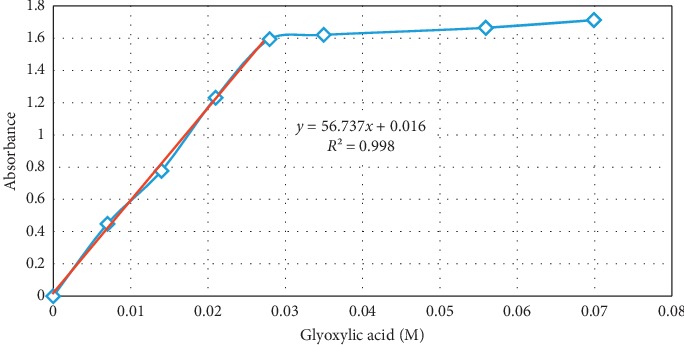
Standard curve for glyoxylic acid and optical absorption in relation to glyoxylic acid concentration at optimized conditions, 0.6 mL tryptophan 0.016 M, 2 mL ferric chloride 0.025 M, and 5 mL concentrated sulphuric acid.

The standard calibration curve was prepared for glyoxylic acid concentration according to the optimized procedure conditions. The spectrophotometer was set zero using the blank sample that comprised water instead of glyoxylic acid in the test mixture.  Figure 8 shows the relation between absorbance and glyoxylic acid concentration at 560 nm. A linear relation between color intensity and glyoxylic acid concentration was obtained in the range 0–0.028 M representative for the calibration curve for glyoxylic acid. At the point 0.028 M, it is supposed that glyoxylic acid amount (0.7 × 10^−5^ moles) reacts with the corresponding stoichiometric amount of tryptophan (0.48 × 10^−5^ moles from Figure 7). The further slight increase in absorbance afterwards can be ascribed to interaction of the excess of glyoxylic acid with tryptophan's secondary products with the iron II ion.

## 4. Mechanism Discussion

**Figure 9:**
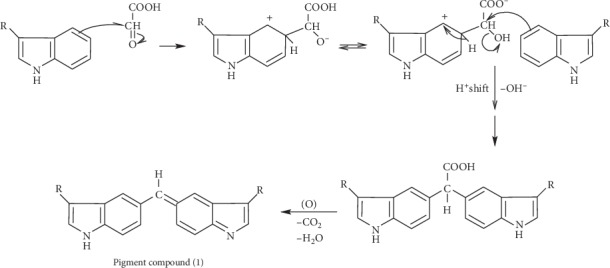
Proposed reaction mechanism of condensation reaction involving two tryptophan molecules and one molecule of glyoxylic acid.

In the early stage of Hopkins–Cole test discovery, it was confirmed that tryptophan is capable to give two distinct colored products when reacted with glyoxylic acid [13]: with the minimum amount of the aldehyde and temperature kept below 15°C, the first colored product is carmine. The mechanism of its formation is assumed to follow the scheme postulated in Figure 9. Here, the aldehyde unites the carbon atoms of the benzene rings of two tryptophan molecules to form the leuco compound, and this upon oxidation forms the quinonoid configuration in the molecule responsible of the color (pigment compound (1)); the same product that appears in equation (1). Such suggested pathway requires the presence of a strong condensing agent like concentrated H_2_SO_4_ or pure acetic acid and a mild oxidant like Fe_aq_^+3^.

**Figure 10:**
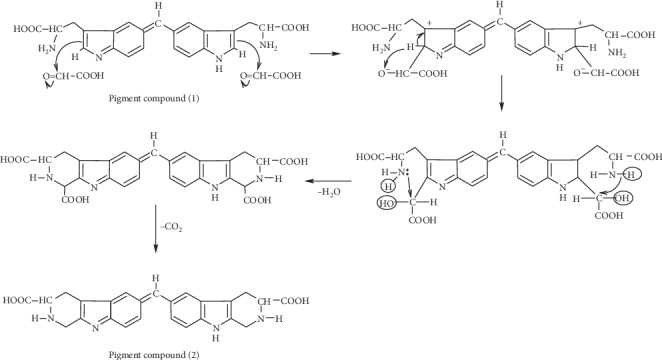
Proposed reaction mechanism of condensation involving two molecules of tryptophan and three molecules of glyoxylic acid.

By allowing the temperature to rise to around 50°C, sufficient activation energy would be available to trigger further condensation steps leading to the second colored product (pigment compound (2)) that appears from violet to blue, depending on water content, blue only in its solid form. Thus, the firstly formed pigment compound (1) condenses further with aldehydes of two more glyoxylic acids. This condensation, nevertheless, occurs between the nitrogen of side chain and the carbon atom of the indole nucleus in the same tryptophan molecule. It leads consequently to closure of the tryptophan side chain and formation of a pyridine nucleus [13]. Such colored product might be formed according to the pathway speculated in Figure 10. It also absorbs at the same wavelength, 560 nm due to alike quinonoid configuration in the molecule responsible of the color.

Friedman and Finley [27] proposed another reaction mechanism between glyoxylic acid and tryptophan in presence of a reducing agent, NaNO_2_, in which glyoxylic acid unites the carbon atoms of the indole nucleus instead of benzene rings of two tryptophan molecules to form the leuco compound.

In our work, we calculated the reaction stoichiometry based on Figures 7 and 8. It is found that the reaction stoichiometry of tryptophan to glyoxylic acid is 2 : 3, thus confirming the proposed mechanism.

Not all aldehydes seem to react with tryptophan because glyoxal gave a negative test result. It appears that only aldehydes that react readily with the benzene ring, indole nucleus, or amino group are capable of condensing with tryptophan.

## 5. Validation of the Method

To validate our method, we measured glyoxylic acid in two organic mixtures comprising the studied compounds with known concentrations using this method and a reference HPLC method after [21]. The results are shown in Tables 1 [Table tab2]–3. The spectrophotometric method achieved comparable results with the selected HPLC method in terms of lower standard deviation, relative standard deviation, and better recovery. Glyoxylic acid can be determined by the proposed spectrophotometric method in more diluted solutions as noticed from LOD and LOQ values. Both methods however ensure same precision and same credibility based on *F*- and *t*-statistical tests, respectively, because both exhibit calculated test values smaller than critical one.

## 6. Conclusions

Glyoxylic acid has been determined in an organic mixture consisting of oxalic acid, glyoxylic acid, glycolic acid, acetic acid, glyoxal, and ethylene glycol by a new spectrophotometric method based on its reaction with tryptophan in presence of ferric chloride and concentrated sulphuric acid. The new method does not require prior separation of glyoxylic acid from the mixture. The reaction between tryptophan and glyoxylic acid was found to be affected by reaction conditions. Good control of temperature and heat removal and correct reagent's amounts are critical for success of the new method. The method was found to be effective within the range 0.00557–0.028 M of glyoxylic acid concentration. The limit of detection, limit of quantitation, standard deviation, relative standard deviation, and recovery of the proposed method were found comparable with a selected HPLC reference method. Both methods displayed same precision and credibility. Reaction stoichiometry of tryptophan to glyoxylic acid was found to be 2 : 3, and the product is tryptophan blue dye. Although it is a dialdehyde, glyoxal did not react with tryptophan according to the Hopkins–Cole test.

## Figures and Tables

**Table 1:** Comparison between spectrophotometric and HPLC methods in terms of standard deviation, recovery%, and RSD%.

Method	Glyoxylic acid concentration in the mixture M	Determined glyoxylic acid in the mixture M^*∗*^	Standard deviation ± M	Recovery (%)	RSD (%)
Spectrophotometric	0.35	0.354	0.00132	101	0.37
	0.50	0.504	0.00110	109	0.22

HPLC	0.35	0.361	0.00466	103	1.29
	0.50	0.508	0.00457	102	0.90

^*∗*^Samples diluted 25 times in the spectrophotometric determination only. RSD: relative standard deviation.

**Table 2 tab2:** Statistical comparison between spectrophotometric and HPLC methods.

Method	Linear regression equation	Correlation coefficient *R* ^2^	LOD (M)	LOQ (M)
Spectrophotometric	*y* = 56.737*x* + 0.016	0.998	0.001905	0.005773
HPLC	*y* = 24337025.58*x* + 219631.2716	0.997	0.052893	0.160282

LOD: limit of detection. LOQ: limit of quantitation.

**Table 3:** Statistical comparison between spectrophotometric and HPLC methods (continued).

Method	*F*-test ^*∗*^calculated	*F*-test critical^*∗∗*^	*t*-test ^*∗*^calculated	*t*-test critical^*∗∗*^
Spectrophotometric	12	19	1.35	4.3
HPLC				

^*∗*^ *F*- and *t*-tests were calculated using measurement data of 0.35 M GA concentration, with 2 degrees of freedom, and 95% confidence level for both series. ^*∗∗*^According to statistical tables given in [28].

## Data Availability

The data used to support the findings of this study are available from the corresponding author upon request.

## Supplementary Materials

Supplementary Materials Graphical summary shows in the upper section the procedure steps and in the bottom section the proposed condensation reaction between 2 tryptophan and 3 glyoxylic acid molecules to form the violet-colored product that is used to determine glyoxylic acid.
