# Supplementary material for: A Simple Spectrophotometric Method for Determination of Glyoxylic Acid in Its Synthesis Mixture
Source: Int J Anal Chem. 2020 Mar 19;2020:5417549. doi: 10.1155/2020/5417549 (PMC7155763; doi:10.1155/2020/5417549)
Supplement: Supplementary Materials — Graphical summary shows in the upper section the procedure steps and in the bottom section the proposed condensation reaction between 2 tryptophan and 3 glyoxylic acid molecules to form the violet-colored product that is used to determine glyoxylic acid. [file 5417549.f1.docx]

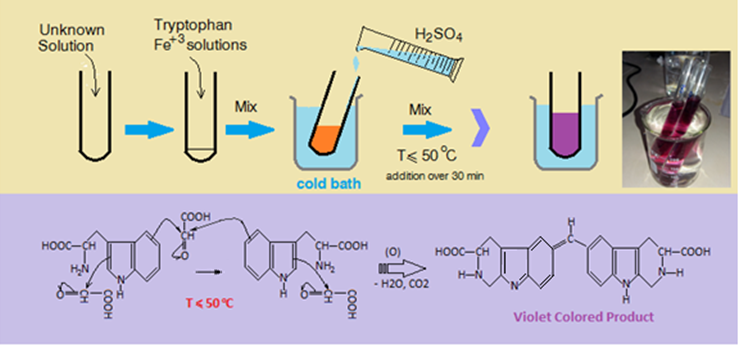


**Graphical Summary** showing in the upper section the procedure steps and in the bottom section the proposed condensation reaction between 2 tryptophan and 3 glyoxylic acid molecules to form the violet colored product that is used to determine glyoxylic acid
